# Evaluation of clinical prediction models (part 2): how to undertake an external validation study

**DOI:** 10.1136/bmj-2023-074820

**Published:** 2024-01-15

**Authors:** Richard D Riley, Lucinda Archer, Kym I E Snell, Joie Ensor, Paula Dhiman, Glen P Martin, Laura J Bonnett, Gary S Collins

**Affiliations:** 1 Institute of Applied Health Research, College of Medical and Dental Sciences, University of Birmingham, Birmingham B15 2TT, UK; 2 National Institute for Health and Care Research (NIHR) Birmingham Biomedical Research Centre, Birmingham, UK; 3 Centre for Statistics in Medicine, Nuffield Department of Orthopaedics, Rheumatology and Musculoskeletal Sciences, University of Oxford, Oxford, UK; 4 Division of Informatics, Imaging and Data Science, Faculty of Biology, Medicine and Health, University of Manchester, Manchester Academic Health Science Centre, Manchester, UK; 5 Department of Biostatistics, University of Liverpool, Liverpool, UK

## Abstract

External validation studies are an important but often neglected part of prediction model research. In this article, the second in a series on model evaluation, Riley and colleagues explain what an external validation study entails and describe the key steps involved, from establishing a high quality dataset to evaluating a model’s predictive performance and clinical usefulness.

A clinical prediction model is used to calculate predictions for an individual conditional on their characteristics. Such predictions might be of a continuous value (eg, blood pressure, fat mass) or the probability of a particular event occurring (eg, disease recurrence), and are often in the context of a particular time point (eg, probability of disease recurrence within the next 12 months). Clinical prediction models are traditionally based on a regression equation but are increasingly derived using artificial intelligence or machine learning methods (eg, random forests, neural networks). Regardless of the modelling approach, part 1 in this series emphasises the importance of model evaluation, and the role of external validation studies to quantify a model’s predictive performance in one or more target population(s) for model deployment.^1^ Here, in part 2, we describe how to undertake such an external validation study and guide researchers through the steps involved, with a particular focus on the statistical methods and measures required, complementing other existing work.^2^ ^3^ ^4^ ^5^ ^6^ ^7^ ^8^ ^9^ ^10^ ^11^ ^12^ ^13^ These steps form the minimum requirement for external validation of any clinical prediction models, including those based on artificial intelligence, machine learning or regression.

**Summary points**

External validation is the evaluation of a model’s predictive performance in a different (but relevant) dataset, which was not used in the development process
An external validation study involves five key steps: obtaining a suitable dataset, making outcome predictions, evaluating predictive performance, assessing clinical usefulness, and clearly reporting findings
The validation dataset should represent the target population and setting in which the model is planned to be implemented
At a minimum, the validation dataset must contain the information needed to apply the model (ie, to make predictions) and make comparisons to observed outcomes
A model’s predictive performance should be examined in terms of overall fit, calibration, and discrimination, in the overall population and ideally in key subgroups (eg, defined by ethnic group), as part of fairness checks
Calibration should be examined across the entire range of predicted values, and at each relevant time point for which predictions are being made, using a calibration plot including a smoothed flexible calibration curve
Where the goal is for predictions to direct decision making, a prediction model should also be evaluated for its clinical usefulness, for example, using net benefit and decision curves
Although a well calibrated model is ideal, a miscalibrated model might still have clinical usefulness
The TRIPOD (Transparent Reporting of a multivariable prediction model for Individual Prognosis Or Diagnosis) statement provides guidance on how to report external validation studies

## What do we mean by external validation?

External validation is the evaluation of a model’s predictive performance in a different (but relevant) dataset, which was not used in the development process.^1^ ^5^ ^7^ ^14^ ^15^ ^16^ ^17^ ^18^ It does not involve refitting the model to compare how the refitted model equation (or its performance) changes compared to the original model. Rather, it involves applying a model as originally specified and then quantifying the accuracy of the predictions made. Five key steps are involved: obtaining a suitable dataset, making outcome predictions, evaluating predictive performance, assessing clinical usefulness, and clearly reporting findings. In this article, we outline these steps, using real examples for illustration.

## Step 1: Obtaining a suitable dataset for external validation

The first step of an external validation study is obtaining a suitable, high quality dataset.

### What quality issues should be considered in an external validation dataset?

A high quality dataset is more easily attained when initiating a prospective study to collect data for external validation, but this approach is potentially time consuming and expensive. The use of existing datasets (eg, from electronic health records) is convenient and often cheaper but is of limited value if the quality is low (eg, predictors are missing, outcome or predictor measurement methods do not reflect actual practice, or time of event is not recorded). Also, some existing datasets have a narrower case mix than the wider target population owing to specific entry criteria; for instance, UK Biobank is a highly selective cohort, restricted to individuals aged between 40 and 69—therefore, its use for external validation would leave uncertainty about a model’s validity for the wider population (including those aged <40 or >69).

To help judge whether an existing dataset is suitable for use in an external validation study, we recommend using the signalling questions within the Prediction model Risk Of Bias ASsessment Tool (PROBAST) domains for Participant Selection, Predictors and Outcome (box 1).^19^ ^20^ Fundamentally, the dataset should be fit for purpose, such that it represents the target population, setting, and implementation of the model in clinical practice. For instance, it should have patient inclusion and exclusion criteria that match those in the target population and setting for use (eg, in the UK, prediction models intended for use in primary care might consider databases such as QResearch, Clinical Practice Research Datalink, Secure Anonymised Information Linkage, and The Health Improvement Network); measure predictors at or before the start point intended for making predictions; ensure measurement methods (for predictors and outcomes) reflect those to be used in practice; and have suitable follow-up information to cover the time points of interest for outcome prediction. It should also have a suitable sample size to ensure precise estimates of predictive performance (see part 3 of our series),^21^ and ideally the amount of missing data should be small (see section on dealing with missing data, below).

Box 1 **Signalling questions within the first three domains of PROBAST (Prediction model Risk Of Bias ASsessment Tool)^19^ ^20^ that are important to consider when ensuring a dataset for external validation is fit for purpose**

**Domain 1: participant selection**

- Were appropriate data sources used—for example, cohort or randomised trial for prognostic prediction model research, or cross sectional study for diagnostic prediction model research?
- Were all inclusions and exclusions of participants appropriate?

**Domain 2: predictors**

- Were predictors defined and assessed in a similar way for all participants?
- Were predictor assessments made without knowledge of outcome data?
- Are all predictors available at the time the model is intended to be used?

**Domain 3: outcome**

- Was the outcome determined appropriately?
- Was a prespecified or standard outcome definition used?
- Were predictors excluded from the outcome definition?
- Was the outcome defined and determined in a similar way for all participants?
- Was the outcome determined without knowledge of predictor information?
- Was the time interval between predictor assessment and outcome determination appropriate?

### What population and setting should be used for external validation of a prediction model?

Researchers should focus on evaluating a model’s target validity,^1^ ^3^ such that the validation study represents the target population and setting in which the model is planned to be implemented (otherwise it will have little value). The validation study might include the same populations and settings that were used to develop the model. However, it could be a deliberate intention to evaluate a model’s performance in a different target population (eg, country) or setting (eg, secondary care) than that used in model development. For this reason, multiple external validation studies are conducted for the same model, to evaluate performance across different populations and settings. For example, the predictive performance of the Nottingham Prognostic Index has been evaluated in many external validation studies.^22^ The more external validations that confirm good performance of a model in different populations and settings, the more likely it will be useful in untested populations and settings.

Most external validation studies are based on data that are convenient (eg, already available from a previous study) or easy to collect locally. As such, they often only evaluate a model’s performance in a specific target setting or (sub)population. To help clarify the scope of the external validation, Debray et al^5^ recommend that researchers should quantify the relatedness between the development and validation datasets, and to make it clear whether the focus of the external validation is on reproducibility or transportability. Reproducibility relates to when the external validation dataset is from a population and setting similar to that used for model development. Reproducibility is also examined when applying internal validation methods (eg, cross validation, bootstrapping) to the original development data during the model development, as discussed in our first paper.^1^ Conversely, transportability relates to external validation in an intended different population or setting, for which model performance is often expected to change owing to possible differences in predictor effects and the participant case mix compared with the original development dataset (eg, when moving from a primary care to a secondary care setting).

### What information needs to be recorded in the external validation dataset?

At a minimum, the external validation dataset must contain the information needed to apply the model (ie, to make predictions) and make comparisons to observed outcomes. This required information means that, for each participant, the dataset should contain the outcome of interest and the values of any predictors included in the model. For time-to-event outcomes, any censoring times (ie, end of follow-up) and the time of any outcome occurrence should also be recorded. Fundamentally, the outcome should be reliably measured, and the recorded predictor information must reflect how, and the moment when, the model will be deployed in practice. For example, for a model to be used before surgery to predict 28 day mortality after surgery, it should use predictors that are available before surgery, and not any perioperative or postoperative predictors.

## Step 2: Making predictions from the model

**Fig 1:**
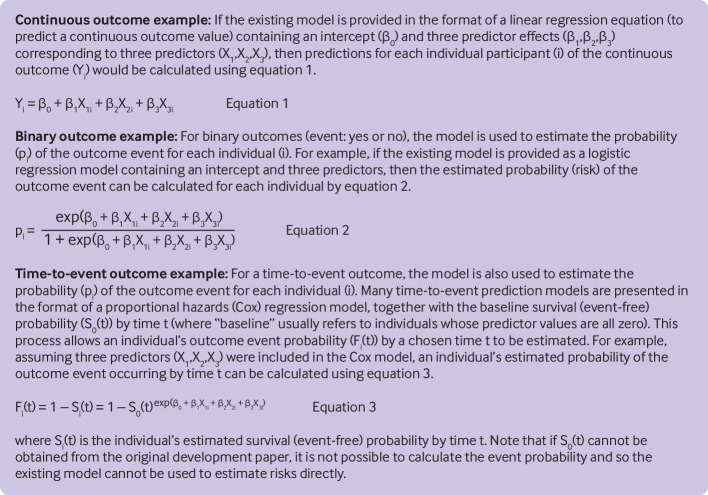
Application of an existing prediction model to derive predicted values for each participant in the external validation dataset

**Fig 2:**
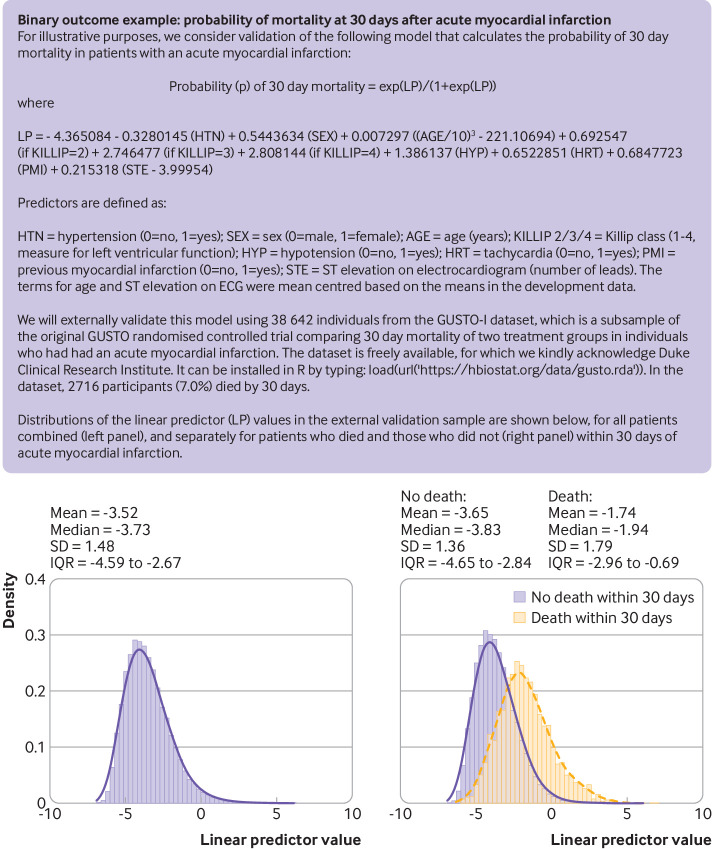
Example of a binary outcome prediction model to be externally validated in new data. SD=standard deviation; IQR=interquartile range

**Fig 3:**
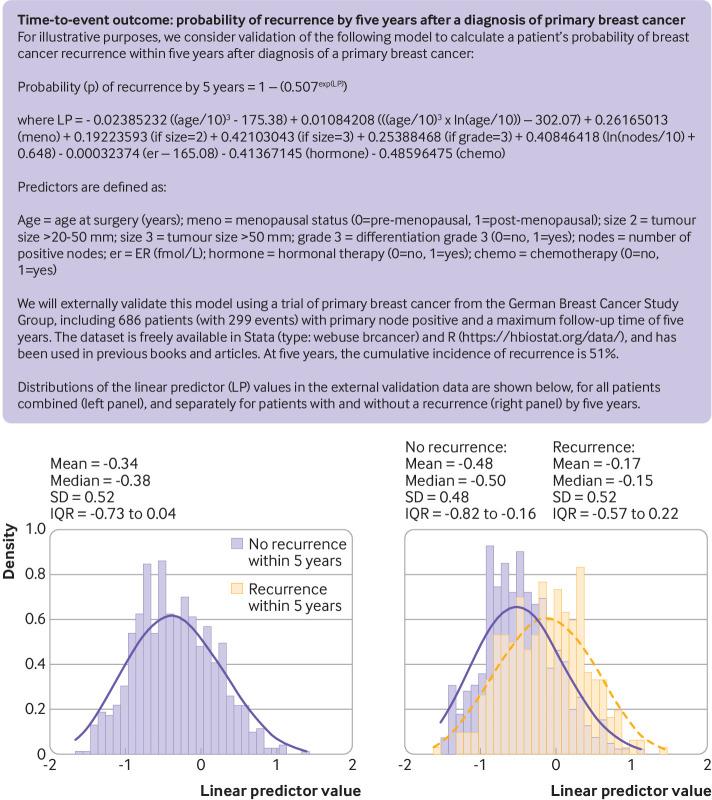
Example of a time-to-event outcome prediction model to be externally validated in new data. SD=standard deviation; IQR=interquartile range

Once the external validation dataset is finalised (ready for analysis), the next step is to apply the existing prediction model to derive predicted values for each participant in the external validation dataset. This step should not be done manually, but rather done by using appropriate (statistical) code that can be programmed to apply the model to each participant in the external validation dataset and compute predicted outcome values based on their multiple predictor values. For some models, typically those based on black box artificial intelligence or machine learning methods, by design they can only be made directly available (by the model developers) as a software object, or accessible via a specific system or server. Figure 1 illustrates the general format of using regression based prediction models to estimate outcome values or event probabilities (risks), and figure 2 and figure 3 provide two case studies.

Figure 2 shows a prediction model developed using the US West subset of the GUSTO-I data (2188 individuals, 135 events), which estimates the probability of 30 day mortality after an acute myocardial infarction.^23^ The logistic regression model includes eight predictors (with 10 predictor parameters, since an additional two parameters are required to capture the categorical Killip classification). For illustration of externally validating this model, we use the remaining data from the GUSTO-I dataset (with thanks to Duke Clinical Research Institute),^23^ which contains all eight predictor variables and outcome information for 38 642 individuals.

Figure 3 shows a prediction model for calculating the five year probability of a recurrence in patients with a diagnosis of primary breast cancer. This survival model was developed for illustrative purposes in 1546 (node positive) participants (974 events) from the Rotterdam breast cancer study,^18^ ^24^ including eight predictors with 10 predictor parameters. External validation is carried out using data from the German Breast Cancer Study Group, which contains all eight predictor variables and outcome information for 686 patients (with 299 events).^18^ ^25^ ^26^ ^27^

Once the predictions have been calculated for each participant, it is good practice to summarise their observed distribution, for example, as a histogram, with summary statistics such as the mean and standard deviation. This presentation is illustrated for the two examples in figure 2 and figure 3, separately for those individuals with and without the outcome event.

## Step 3: Quantifying a model’s predictive performance

The third step is to quantify a model’s predictive performance in terms of overall fit, calibration, and discrimination. This step requires suitable statistical software, which is discussed in supplementary material S1,^28^ ^29^ ^30^ ^31^ ^32^ and example code is provided at www.prognosisresearch.com/software. 

### Overall fit

Overall performance of a prediction model for a continuous outcome is quantified by R^2^, the proportion of the total variance of outcome values that is explained by the model, with values closer to 1 preferred. Often this value is multiplied by 100, to give the percentage of variation explained. Generalisations of R^2^ for binary or time-to-event outcomes have also been proposed, such as the Cox-Snell R^2^ (this has a maximum value below 1),^33^ Nagelkerke’s R^2^ (a scaled version of the Cox-Snell R^2^, which has a maximum value of 1),^34^ O’Quigley’s R^2^,^35^ Royston’s R^2^,^36^ and Royston and Sauerbrei’s R^2^_D_.^37^ We particularly recommend reporting the Cox-Snell R^2^ value, as it is needed in sample size calculations for future model development studies.^38^

Another overall measure of fit is the mean squared error of predictions, which for continuous outcomes can be obtained on external validation by calculating the mean of the squared difference between participants’ observed outcomes and their estimated (from the model) outcomes. An extension of the mean square error for binary or time-to-event outcomes is the Brier score,^39^ ^40^ which compares observed outcomes and estimated probabilities. Overall fit performance estimates are shown for the two examples in table 1.

**Table 1:** Predictive performance of example models when examined in the external validation population. Data are estimates (95% confidence intervals). AUROC=area under the receiver operating characteristic curve

Performance measure	Binary outcome model: probability of 30 day mortality after an acute myocardial infarction	Time-to-event outcome model: probability of five year recurrence after a diagnosis of primary breast cancer
**Overall fit **		
R^2^ Nagelkerke	0.077 (0.070 to 0.085)	—
R^2^ Cox-Snell	0.19 (0.18 to 0.21)	—
Royston R^2^_D_	—	0.17 (0.12 to 0.23)
Brier score	0.059 (0.057 to 0.061)	0.22 (0.19 to 0.24)
**Calibration**		
Calibration-in-the-large	0.019 (−0.026 to 0.064)	—
Observed/expected	1.01 (1.01 to 1.02)	1.27 (1.22 to 1.32)
Calibration slope	0.72 (0.70 to 0.75)	1.10 (0.88 to 1.33)
Integrated calibration index	0.017 (0.017 to 0.018)	0.109 (0.107 to 0.112)
**Discrimination **		
C statistic (AUROC)	0.81 (0.80 to 0.82)	—
Harrell’s C index	—	0.67 (0.64 to 0.70)
Time dependent AUROC at five years	—	0.71 (0.65 to 0.76)

### Calibration plots

**Fig 4:**
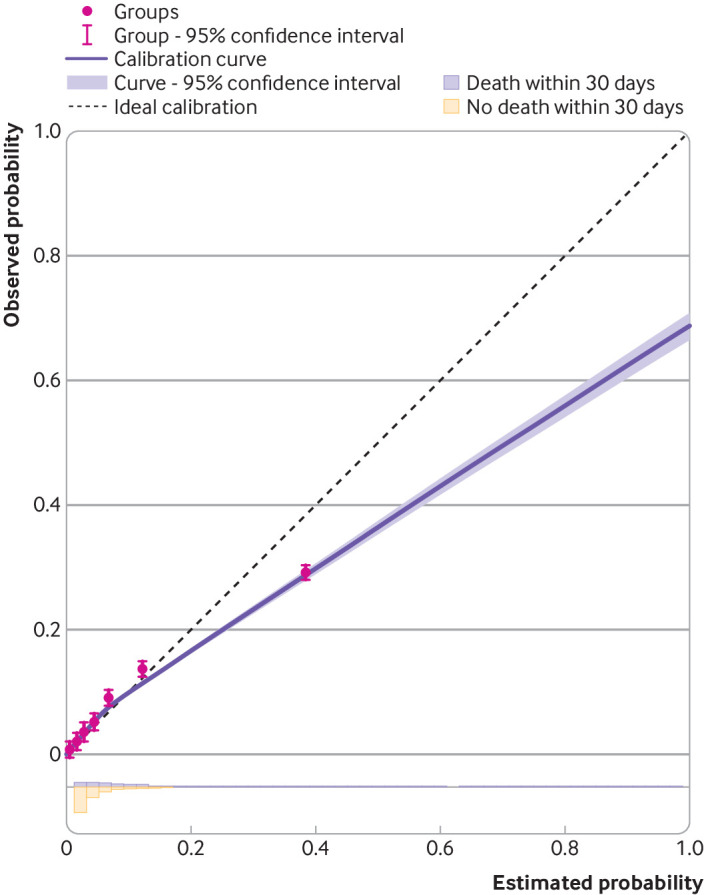
Calibration plots for binary outcome prediction model on external validation. Example shows probability of 30 day mortality after an acute myocardial infarction. Area below the dashed line=where the model’s risk estimates are too high; area above the dashed line=where the model’s risk estimates are too low; 10 circles=10 groups defined by tenths of the distribution of estimated risks; histograms at the bottom of graphs show the distribution of risk estimates for each outcome group

**Fig 5:**
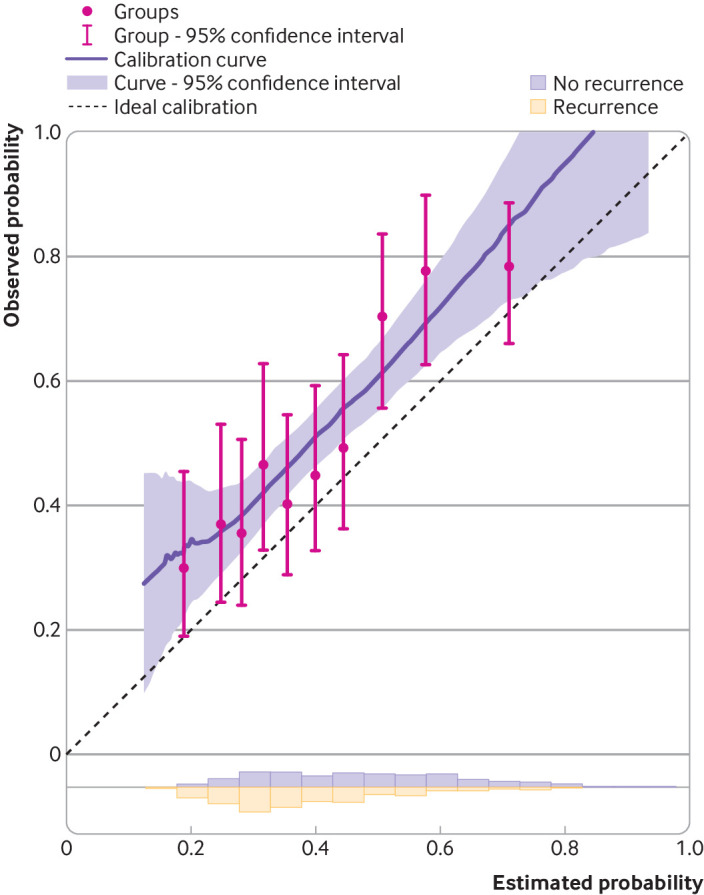
Calibration plots for time-to-event prediction model on external validation. Example shows time-to-event outcome: probability of five year recurrence after a diagnosis of primary breast cancer. Area below the dashed line=where the model’s risk estimates are too high; area above the dashed line=where the model’s risk estimates are too low; 10 circles=10 groups defined by tenths of the distribution of estimated risks; histograms at the bottom of graphs show the distribution of risk estimates for each outcome group

Calibration refers to the assessment of whether observed and predicted values agree.^41^ For example, whether observed event probabilities agree with a model’s estimated event probabilities (risks). Although an individual’s event probability cannot be observed (we only know if they had the outcome event or not), we can still examine calibration of predicted and observed probabilities by deriving smoothed calibration curves fitted using all the individuals’ observed outcomes and the model’s estimated event probabilities (fig 4 and fig 5). At external validation, some miscalibration between the predicted and observed values should be anticipated. The more different the validation dataset is compared with the development dataset (eg, in terms of population case mix, outcome event proportion, timing and measurement of predictors, outcome definition), the greater the potential for miscalibration. Similarly, models developed using low quality approaches (eg, small datasets, unrepresentative samples, unpenalised rather than penalised regression) have greater potential for miscalibration on external validation.

Calibration should be examined across the entire range of predicted values (eg, probabilities between 0 to 1), and at each relevant time point for which predictions are being made. Van Calster et al outline a hierarchy of calibration checks,^42^ ranging from the overall mean to subgroups defined by patterns of predictor values. Fundamentally, calibration should be visualised graphically using a calibration plot that compares observed and predicted values in the external validation dataset, and the plot must include a smoothed flexible calibration curve (with a confidence interval) as fitted in the individual data using a smoother or splines.^42^ ^43^

Many researchers, however, do not report a calibration plot,^44^ and those that do tend to only report grouped points rather than a calibration curve across the entire range. Grouping can be gamed (eg, by altering the number of groups), only reveals calibration in the ill defined groups themselves, and caps the calibration assessment at the average predicted value in the lowest and highest group. Hence, grouping enables researchers to (deliberately) obfuscate any meaningful assessment of miscalibration in particular ranges of predicted values (an example is shown below). A calibration curve provides a more complete picture. For continuous outcomes, the calibration plot and smoothed curve can be supplemented by presenting the pair of observed (y axis) against predicted (x axis) values for all participants. For binary or time-to-event outcomes, observed (y axis) event probabilities against the model’s estimated event probabilities (x axis) can be added for groups defined by, for example, 10ths or 20ths of the model’s predictions—again, to supplement (not replace) a smoothed calibration curve.^43^

The calibration plot should be presented in a square format, and the axes should not be distorted (eg, by changing the scale of one of the axes, or having uneven spacing across the range of values) as this could hide miscalibration in particular regions. Researchers should also add the distribution of the predicted values underneath the calibration plot, to show the spread of predictions in the validation dataset, perhaps even for each of the event and non-event groups separately.

If censoring occurs before the time point of interest in the validation dataset, then the true outcome event status is unknown for the censored individuals, which makes it difficult to directly plot the calibration of model predictions at the time point of interest. A common approach is to create groups (eg, 10 groups defined by tenths of the model’s estimated event probabilities), and to plot the model’s average estimated probability against the observed (1–Kaplan-Meier) event probability for each group. However, this approach is unsatisfactory, because the number of groups and the thresholds used to define them are arbitrary; hence, it only provides information on subjectively chosen groups of participants and does not provide granular information on calibration or miscalibration at specified values or ranges of predicted values. To manage this problem, a smoothed calibration curve can be plotted that examines calibration across the entire range of predicted values (analogous to the calibration plot for binary outcomes) at a particular time point. This approach can be achieved using pseudo-observations (or pseudo-values),^45^ ^46^ ^47^ ^48^ or flexible adaptive hazard regression or a Cox model using restricted cubic splines.^49^ More details are provided in supplementary material S2.

Calibration plots and curves for the two examples are shown in figure 4 and figure 5. The calibration plot for the binary outcome example (fig 4) shows good calibration for event probabilities between 0 and 0.15. For calculated event probabilities beyond 0.2, the model overestimates the probability of mortality, as revealed by the smoothed calibration curve lying below the diagonal line. Had only grouped points been included (and not a smoothed curve across individuals), the extent of the miscalibration in the range of model predictions above 0.2 would be hidden. For example, consider if the calibration had been checked for 10 groups based on tenths of predicted values (see 10 circles in fig 4). Because most of the data involve patients with model predictions less than 0.2, nine of the 10 groups fall below predictions of 0.2. Further, the model’s estimated probabilities in the upper group have a mean of about 0.4, and information above this value is completely lost, incidentally where the miscalibration is most pronounced based on the smoothed curve across all individuals. Therefore, figure 4 demonstrates our earlier point that categorising into groups loses and hides information, and that the calibration curve is essential to show information across the whole range of predictions, including values close to 1.

Although a well calibrated model is ideal, a miscalibrated model might still have clinical usefulness. For example, in figure 4, miscalibration is most pronounced in regions where the model’s estimated mortality risks are very high (eg, >0.3), with actual observed risks about 0.05 to 0.3 lower. However, in this setting, whether a patient is deemed to have high or very high mortality risks is unlikely to change clinical decisions for that patient. By contrast, in regions where clinical risk thresholds are more relevant (eg, predictions ranging from 0.05 to 0.1), calibration is very good and so the model might still be useful in clinical practice despite the miscalibration at higher risks (see step 4).

The calibration plot for the time-to-event outcome example shows that the predictions are systematically lower than the observed event risk at five years (fig 5), with most of the calibration curve lying above the diagonal. In particular, for predictions between 0.1 and 0.8, the model appears to systematically underestimate the probability of recurrence within five years of a breast cancer diagnosis.

The calibration curve’s confidence interval is important to reveal the precision of the calibration assessment. It also quantifies the uncertainty of the actual risk in a group of individuals defined by a particular predicted value. For example, for the group of individuals with an estimated risk of 0.8 in figure 5, the 95% confidence interval around the curve suggests that this group’s actual risk is likely between 0.78 to 1.

### Quantifying calibration performance

Calibration plots with calibration curves should also be supplemented with statistical measures that summarise the calibration performance observed in the plot.^50^ Calibration should not be assessed using the Hosmer-Lemeshow test, or related ones like the Nam-D’Agostino test or Gronnesby-Borgan test, because these require arbitrary grouping of participants that, along with sample size, can influence the calculated P value, and does not quantify the actual magnitude or direction of any miscalibration. Rather, calibration should be quantified by the calibration slope (ideal value of 1), calibration-in-the-large (ideal value of 0) and—for binary or time-to-event outcomes—the observed/expected (O/E) ratio (ideal value of 1) or conversely the E/O ratio. A detailed explanation for each of these measures is given in supplementary material S3. Estimates of these measures should be reported alongside confidence intervals, and derived for the dataset as a whole and, ideally, also for key subgroups (eg, different ethnic groups, regions). To quantify overall miscalibration based on the calibration curve, the estimated or integrated calibration index can be used, which respectively measure an average of the squared or absolute differences between the estimated calibration curve and the 45 degree (diagonal) line of ideal calibration.^51^ ^52^

Calibration measures are summarised in table 1 for the two examples, which confirm the visual findings in the calibration plots. For example, the binary outcome prediction model has a calibration slope of 0.72 (95% confidence interval 0.70 to 0.75), suggesting that predictions are too extreme; this is driven by the overprediction in those with estimated event probabilities above 0.2 (fig 4). The time-to-event prediction model has an O/E ratio of 1.27, suggesting that the observed event probabilities are systematically higher than the model’s estimated values, which is seen by the smoothed calibration curve lying mainly above the diagonal line (fig 5). Such situations could motivate model updating to improve calibration performance.^53^

The results also emphasise how one calibration measure alone does not provide a full picture. For example, the calibration slope is close to 1 for the time-to-event prediction model (1.10, 95% confidence interval 0.88 to 1.33), but there is clear miscalibration owing to the O/E ratio of 1.27 (1.22 to 1.32). Conversely, O/E ratio is 1.01 (1.01 to 1.02) in the binary outcome example, suggesting good overall agreement, but the calibration slope is 0.72 (0.70 to 0.75) owing to the overestimation of high risks (fig 4). Hence, all measures of calibration should be reported together and—fundamentally—alongside a calibration plot with a smoothed calibration curve.

### Quantifying discrimination performance

Discrimination refers to how well a model’s predictions separate between two groups of participants: those who have (or develop) the outcome and those who do not have (or do not develop) the outcome. Therefore, discrimination is only relevant for prediction models of binary and time-to-event outcomes, and not continuous outcomes.

Discrimination is quantified by the concordance (c) statistic (index),^11^ ^54^ and a value of 1 indicates the model has perfect discrimination, while a value of 0.5 indicates the model discriminates no better than chance. For binary outcomes, it is equivalent to the area under the receiver operating characteristic curve (AUROC) curve. It gives the probability that for any randomly selected pair of participants, one with and one without the outcome, the model assigns a higher probability to the participant with the outcome. What constitutes a high c statistic is context specific; in some fields where strong predictors exist, a c statistic of 0.8 might be considered high, but in others where prediction is more difficult, values of 0.6 might be deemed high. The c statistic also depends on the case mix distribution. Presenting an ROC curve over and above the c statistic (AUROC) has very little, if any, benefit.^55^ ^56^ Similarly, providing traditional measures of test accuracy such as sensitivity and specificity are not as relevant for prediction models, because the focus should be on the overall performance of the model’s predictions without forcing thresholds to define so-called high and low groups. If thresholds are important for clinical decision making, then clinical utility should be assessed at those thresholds, for example, using net benefit and decision curves (see step 4).

Generalisations of the c statistic have been proposed for time-to-event models, most notably Harrell’s C index, but many other variants are available, including Efron’s estimator, Uno’s estimator, Göner and Heller’s estimator, and case mix adjusted estimates.^54^ ^57^ Royston’s D statistic is another measure of discrimination,^37^ interpreted as the log hazard ratio comparing two equally sized groups defined by dichotomising the (assumed normally distributed) linear predictor from the developed model at the median value. Higher values for the D statistic indicate greater discrimination.

Harrell’s C index and Royston’s D statistic measure discrimination over all time points up to a particular time point (or end of follow-up). However, usually an external validation study aims to examine a model’s predictive performance at a particular time point, and so time dependent discrimination measures are more informative, such as an inverse probability of censoring weighted estimate of the time dependent area under the ROC curve for the time point of interest (t).^58^

Discrimination performance for the two examples is shown in table 1, and show promising discrimination in both cases. For the binary outcome example, the model correctly identifies 80.8% concordant pairs (c statistic 0.81, 95% confidence interval 0.80 to 0.82). The time-to-event example has a Harrell’s C index of 0.67 (0.64 to 0.70) and a time dependent AUROC curve of 0.71 (0.65 to 0.76), suggesting that the model’s discrimination at five years is slightly higher than the discrimination performance averaged across all time points.

## Step 4: Quantifying clinical utility

Where the goal is for predictions to direct decision making, a prediction model should also be evaluated for its overall benefit on participant and healthcare outcomes; also known as its clinical utility.^16^ ^59^ ^60^ For example, if a model estimates a patient’s event probability above a certain threshold value (eg, >0.1), then the patient and their healthcare professionals could decide on some clinical action (eg, above current clinical care), such as use of a particular treatment, monitoring strategy, or lifestyle change. When externally validating the model, the clinical utility of this approach can be quantified by the net benefit, a measure that weighs the benefits (eg, improved patient outcomes) against the harms (eg, worse patient outcomes, additional costs).^61^ ^62^ It requires the researchers to choose a probability (risk) threshold, at or above which there will be a clinical action. The threshold should be chosen before a clinical utility analysis, based on discussion with clinical experts and patient focus groups, and indeed there might be a range of thresholds of interest, because a single threshold is unlikely to be acceptable for all clinical settings and individuals. Then, a decision curve can be used to display a model’s net benefit across the range of chosen threshold values, and compared with other decision making strategies (eg, other models, or options such as treat all and treat none). Further explanation is provided in supplementary material S4, and more detailed guidance is provided in previous tutorials.^61^ ^62^ ^63^

**Fig 6:**
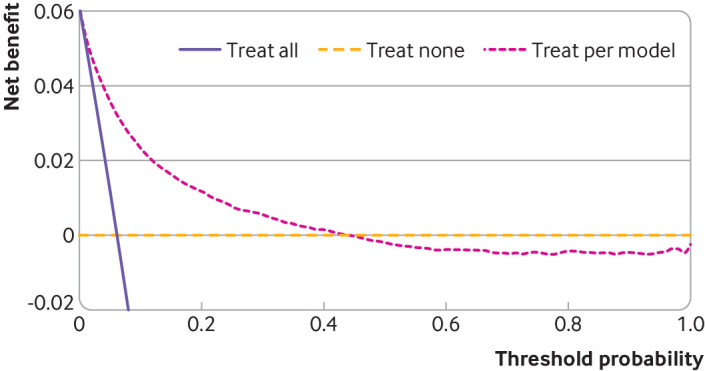
Decision curves showing net benefit for binary outcome prediction model across a range of threshold probabilities that define when some clinical action (eg, treatment) is warranted. Example shows probability of 30 day mortality after an acute myocardial infarction. Threshold probability=risk needed to initiate a particular treatment or clinical action; positive values of net benefit indicate clinical utility; treat all=strategy of initiating the particular treatment (or clinical action) for all patients regardless of their estimated risk; treat none=strategy of not initiating the treatment (or clinical action) for any patient; treat per model=strategy of initiating the treatment (or clinical action) for those patients whose estimated risk is at or above the threshold probability. An interactive version of this graphic is available at: https://public.flourish.studio/visualisation/15175981/

**Fig 7:**
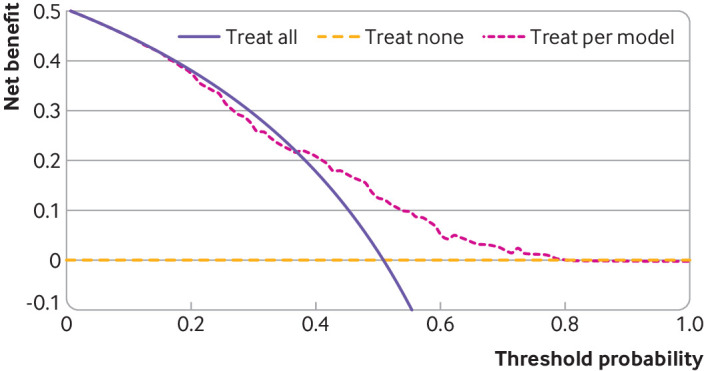
Decision curves showing net benefit for time-to-event prediction model across a range of threshold probabilities that define when some clinical action (eg, treatment) is warranted. Example shows probability of five year recurrence after a diagnosis of primary breast cancer. Threshold probability=risk needed to initiate a particular treatment or clinical action; positive values of net benefit indicate clinical utility; treat all=strategy of initiating the particular treatment (or clinical action) for all patients regardless of their estimated risk; treat none=strategy of not initiating the treatment (or clinical action) for any patient; treat per model=strategy of initiating the treatment (or clinical action) for those patients whose estimated risk is at or above the threshold probability. An interactive version of this graphic is available at: https://public.flourish.studio/visualisation/15162451/

We apply this clinical utility step to the two examples in figure 6 and figure 7, and show results across the entire 0 to 1 probability range for illustration, although in practice a narrower range would be predetermined by clinical and patient groups, as mentioned. Figure 6 shows that the binary outcome model has a positive net benefit for all thresholds below 0.44, where clinical thresholds are likely to fall in this clinical setting, with greater net benefit than the treat all strategy at all thresholds. Figure 7 time-to-event outcome model has a positive net benefit for thresholds up to 0.79, but does not provide added benefit over the treat all strategy if key thresholds fall below 0.38.

## Step 5: Clear and transparent reporting

The Transparent Reporting of a multivariable model for Individual Prognosis Or Diagnosis (TRIPOD) statement provides guidance on how to report studies validating a multivariable prediction model.^50^ ^64^ For example, the guidance recommends specifying all measures calculated to evaluate model performance and, at a minimum, to report calibration (graphically and quantified) and discrimination, along with corresponding confidence intervals. With the introduction of new sample size criteria for both developing and validating prediction models,^21^ ^38^ ^65^ ^66^ ^67^ ^68^ ^69^ ^70^ ^71^ we also recommend reporting either the Cox-Snell or Nagelkerke R^2^, and the distribution of the linear predictor (eg, histograms for those with and without the outcome event, as shown in fig 2 and fig 3, and at the base of the plots in fig 4 and fig 5). These additional reporting recommendations not only provide information on the performance of the model but also provide researchers with key information needed to estimate sample sizes for further external validation, model updating, or when developing new models.^38^ ^65^ ^66^ ^68^

## Special topics

### Dealing with missing data

The external validation dataset might contain missing data in some of the predictor variables or the outcome. A variety of methods are available to deal with missing data, including analysis of complete cases, single imputation (eg, mean or regression imputation), and multiple imputation. Handling of missing data during external validation is an unresolved topic and an area of active research.^72^ ^73^ ^74^ Occasionally the model developers will specify how to deal with missing predictor values during model deployment; in that situation, the external validation should primarily assess that recommended strategy. However, most existing models do not specify or even consider how to deal with missing predictor values at deployment, and an external validation might then need to examine a range of plausible options, such as single or multiple imputation.

### Checking subgroups and algorithmic fairness

An important part of external validation is to check a model’s predictive performance in key clusters (eg, countries, regions) and subgroups (eg, defined by sex, ethnic group), for example, as part of examining algorithm fairness. This is discussed in more detail in paper 1 of our series.^1^

### Multiple external validation studies and individual participant data meta-analyses

Where interest lies in a model’s transportability to multiple populations and settings, multiple external validation studies are often needed.^5^ ^75^ ^76^ ^77^ Then, not only is the overall (average) model performance of interest, but also the heterogeneity in performance across the different settings and populations.^5^ Heterogeneity can be examined through data sharing initiatives and by using individual participant data meta-analyses, as described elsewhere.^4^ ^78^

### Competing events

Sometimes competing events can occur that prevent a main event of interest from being observed, such as death before a second hip replacement. In this situation, if a model’s predictions are to be evaluated in the context of the real world (ie, where the competing event will reduce the probability of the main event from occurring), then the predictive performance estimates must account for the competing event in the statistical analysis (eg, when deriving calibration curves). This topic is covered in a related paper in *The BMJ* on validation of models in competing risks settings.^9^

## Conclusions

External validation studies should be highly valued by the research community. A model is never completely validated,^3^ ^79^ because its predictive performance could change across target settings, populations, and subgroups, and might deteriorate over time owing to improvements in care (leading to calibration drift). Thus, external validation studies should be viewed as a necessary and continual part of evaluating a model’s performance. In the next article in this series, we describe how to calculate the sample size required for such studies.^21^

## Data Availability

The GUSTO-I dataset is freely available, for which we kindly acknowledge Duke Clinical Research Institute. It can be installed in R by typing: load(url('https://hbiostat.org/data/gusto.rda')).

## Web extra

Extra material supplied by authors

Web appendix: Supplementary material

## Notes

Patient and public involvement: Patients or the public were not involved in the design, or conduct, or reporting, or dissemination of our research.

Provenance and peer review: Not commissioned; externally peer reviewed.
