# Supplementary material for: Evaluation of clinical prediction models (part 2): how to undertake an external validation study
Source: BMJ. 2024 Jan 15;384:e074820. doi: 10.1136/bmj-2023-074820 (PMC10788734; doi:10.1136/bmj-2023-074820)
Supplement: Supplementary file 1 — Web appendix: Supplementary material [file rilr074820.ww.pdf]

Supplementary material

S1: Software for calculating predictive performance upon external validation

Within R, a useful package for undertaking external validation of binary and time-to-event models is Harrell's *rms* package.[75] Smoothed calibration plots with pseudo values for time-to-event models are not currently part of the rms package, but can be drawn using the *riskRegression*[76] or *pec* libraries.[77] The package dcurves in R produces decision curves examining net benefit across a range of thresholds (see www.decisioncurveanalysis.org for package details and tutorials).

Stata packages for external validation of clinical prediction models include *pmstats* for calculating a range of performance statistics at external validation, *pmcalplot* for producing calibration plots including smoothed calibration curves,[78] and *dca* for producing decision curves examining net benefit across a range of thresholds (see www.decisioncurveanalysis.org). Each of these packages can be used to validate existing models for binary and time-to-event outcomes (and continuous outcomes using *pm* packages) and require the user to input the outcome variable along with either the published linear predictor or predictions from the existing model calculated for each individual in the external validation dataset.

Note that some measures (such as calibration-in-the-large and overall fit) need software that can constrain parameters to particular values. For example, R^2 measures for a binary outcome prediction model can be obtained on external validation by fitting a logistic regression model for the probability (risk) of the outcome with participants' linear predictor value from the developed model as the only covariate, constraining the intercept term to be zero and the slope to be one. Practically this is achieved in software by setting starting values for these parameter estimates (to zero and one respectively) and reducing the number of iterations in the logistic model calculation to zero.

S2: Smoothed calibration curves in datasets with censoring

Pseudo-observations (or pseudo-values)[39-42] are derived using the jackknife (i.e., leave-one-out) estimator, and produce pseudo-observed event probabilities for each individual. We denote these pseudo-observations by $\tilde{F}_i(t)$ and provided that censoring is independent of covariates, they yield unbiased estimates of the true $F_i(t)$.[40] This allows the generation of a calibration plot at time point t (thereby avoiding grouping) and the derivation of a flexible calibration curve, for example using a smoothing approach.[41, 79] The standard generation of pseudo-observations assumes censoring is uninformative. In practice censoring may be informative (i.e., conditional on covariates and thus underlying event probability), and so to help mitigate against this, we suggest deriving pseudo-observations separately within each of, say, 10 or 20 groups defined by tenths or twentieths of the

model's estimated probabilities. Rather than using pseudo-observations, Austin et al. suggest using flexible adaptive hazard regression or a Cox model using restricted cubic splines, which also allows observed event probabilities to be produced (and thus calibration curves) assuming uninformative censoring conditional on the predicted value and proportional hazards.[43]

S3: Explanation of key measures of calibration for a prediction model with binary or time-to-event outcomes

Observed/Expected ratio (O/E)

O/E summarises the overall calibration. For binary outcomes, it is calculated as the ratio of the total observed to have the outcome event over the total expected to have the outcome event in the prediction time horizon. Thus, an ideal value is 1. Values less than 1 indicate the model is over-predicting the total number of outcomes in the population, whilst values above 1 indicate the model is under-predicting the total number of outcomes in the population. For time-to-event outcomes, mean observed (based on Kaplan-Meier estimates) and expected event probabilities at a specified time point can be used instead of total numbers of outcomes to account for censoring. Note also that sometimes the E/O ratio is presented; under-prediction then occurs for values below 1 and over-prediction for values above 1. For continuous outcomes, an analogous measure of the O/E ratio is the mean predicted outcome value compared to the mean observed outcome value.

Calibration-in-the-large

Calibration-in-the-large is closely related to the overall O/E statistic,[13] but less intuitive to interpret. For binary outcomes, it can be estimated by fitting a logistic model for the probability of the outcome event (p_i) with participants' linear predictor value from the developed model (i.e., $LP_i = \hat{\beta}_0 + \hat{\beta}_1 x_{1i} + \hat{\beta}_2 x_{2i} + \hat{\beta}_3 x_{3i} + \cdots + \hat{\beta}_k x_{ki}$) as a single covariate (as an offset term),

$$\text{logit}(p_i) = \alpha + 1(LP_i)$$

where the estimate of α is the estimate of calibration-in-the-large.[13] Calibration-in-the-large should be close to zero for a well calibrated model. Importantly, calibration-in-the-large may be zero and O/E may be 1 even when there is still substantial miscalibration; that is, on average predictions may appear well calibrated, but there can be under-prediction in some ranges of predicted values which cancels out over-prediction (or vice versa) in other ranges, hence stressing the need for always providing calibration plots in combination with these calibration statistical measures.

Calibration slope

The calibration slope is one measure of agreement between observed and estimated (from the model) event probabilities.[11, 13] When a model is developed using traditional estimation techniques (e.g., unpenalised maximum likelihood estimation), the observed calibration slope will always be 1 in the development dataset. However, upon validation in new data, it will likely deviate from 1. A slope < 1 indicates that in some ranges (which can directly be viewed from the calibration plot) the model predictions are too extreme (i.e., estimated probabilities closer to 1 are too high, and estimated probabilities closer to 0 are too low) and a slope > 1 indicates model predictions are too narrow (i.e., estimated probabilities closer to 1 are too low, and estimated probabilities closer to 0 are too high). A calibration slope < 1 is often observed in external validation studies, consistent with a lack of adjustment for over-fitting (optimism) of the model when it was developed.

To estimate the calibration slope, a model must be fitted in the validation dataset in a similar way as above for the calibration-in-the-large. For binary outcomes, it can be estimated using a logistic regression model with the logit of the observed outcome event probability (p_i) regressed against the linear predictor (LP_i) value as single covariate: $\text{logit}(p_i) = \alpha + \beta LP_i$. Then, $\hat{\beta}$ is the estimated calibration slope. The calibration slope is derived using the LP for each participant and does not require grouping. Similarly, for continuous outcomes is can be derived by fitting a linear regression model of the form $Y_i = \alpha + \beta LP_i$

For time-to-event outcomes, a generalised linear model can be fitted with $\tilde{F}_i(t)$ as the outcome response, and the expected value ($\mathrm{E}\left(\tilde{F}_i(t)\right)$) modelled using a particular link function, such as a logit function or a complementary log-log link function.[41] Model fitting is discussed by Andersen and Perme,[40] who suggest the use of generalised estimating equations followed by a sandwich estimator for the variance of parameter estimates (which is needed to account for the correlation of the pseudo-observations themselves).

S4: An introduction to net benefit and decision curves

The overall consequences of using a prediction model for clinical decisions can be measured using net benefit, which requires only the weighing of the benefits (e.g., improved patient outcomes) against the harms (e.g., worse patient outcomes, additional costs).[55, 56] It requires the researchers to choose a probability (risk) threshold $p_{\text{threshold}}$, such that if an individual's estimated probability of the outcome event (p_i) is $\geq p_{\text{threshold}}$ there will be a clinical action (e.g., onset of treatment, referral to specialist, etc). Based on the chosen threshold, the net benefit is the difference between the number of true-positive (TP) results and the number of false-positive (FP) results, relative to the total sample size (N), and weighted by a factor $\frac{p_{\text{threshold}}}{1-p_{\text{threshold}}}$. The weighting factor is the odds of the outcome event at the chosen threshold value (the probability of the outcome event divided by the probability of not having the outcome event), which can equivalently be considered to represent an acceptable harm to benefit ratio. In other words, the chosen $p_{\text{threshold}}$ should reflect where the expected benefit of clinical action is equal to the expected benefit of avoiding clinical action.[56]

For a prediction model with a binary outcome, net benefit (NB_{p_t}) at a threshold of $p_{\text{threshold}}$ can be calculated as,

$$NB_{p_t} = \frac{TP}{N} - \left(\frac{FP}{N} \times \frac{p_{\text{threshold}}}{1 - p_{\text{threshold}}}\right) = \frac{TP - \left(FP \times \frac{p_{\text{threshold}}}{1 - p_{\text{threshold}}}\right)}{N} \qquad \text{Eq. (3)}$$
$$= (\text{sensitivity} \times \phi) - \left((1 - \text{specificity}) \times (1 - \phi) \times \frac{p_{\text{threshold}}}{1 - p_{\text{threshold}}}\right)$$

where ϕ is the observed outcome event proportion in the entire dataset. Positive values of the net benefit indicate the model has clinical utility, as the benefits outweigh the harms at the specific threshold. It is helpful to multiply the net benefit by 100 (or 1000), so it can be interpreted as the additional number of true cases identified for treatment (or some clinical action) without increasing the number treated unnecessarily per 100 (or 1000) individuals. The net benefit is zero if the benefit compensates the harm, and negative if harm surpasses benefit.

The maximum possible value of the net benefit is the outcome event proportion (ϕ), therefore it is bounded below 1.[55] The standardised net benefit (sNB_{p_t}) is defined as NB_{p_t}/ϕ, and the standardisation ensures that the maximum value is 1 regardless of the validation setting. Often it is helpful to compare a model's (standardised) net benefit to that of a 'treat none' strategy, which is by definition zero. Similarly, a comparison to a 'treat all' strategy can be made, where $NB_{p_t}(\text{treat all}) = \phi - [(1 - \phi) \times \frac{p_{\text{threshold}}}{1-p_{\text{threshold}}}]$.

The Net Benefit calculation has also be extended to time-to-event outcomes.[57] To allow for censoring during follow up, Equation 3 is easily adapted such that the numbers of TP and FP results are derived from estimates of the survival probability at our timepoint of interest $(S(t))$. The number of TP results at a given threshold, $p_{\text{threshold}}$, is calculated by $[1 - S(t) \mid p_i \geq p_{\text{threshold}}]$ x $P(p_i \geq p_{\text{threshold}})$ x N, while the number of FP results is calculated as $[S(t) \mid p_i \geq p_{\text{threshold}}]$ x $P(p_i \geq p_{\text{threshold}})$ x N. The survival function $S(t)$ can be based on Kaplan-Meier estimates or the cumulative incidence function for an event, as appropriate.

The threshold ($p_{\text{threshold}}$) for calculating net benefit should be chosen *before* analysis, based on discussion with clinical experts and patient focus groups, and indeed there may rather be a range of thresholds of interest, as a single threshold is unlikely to be acceptable in all clinical settings and individuals. Then, a decision curve can be used to display the net benefit of using the prediction model across the range of values, and again compare to strategies of treat all and treat none. Application is made to the two motivating examples in **Error! Reference source not found.**, with results shown across the entire 0 to 1 probability range for illustration, though in practice a narrower range will usually be pre-determined based on the clinical and patient groups..

The plot may show a model has net benefit over other strategies (or models) in some regions but not in others – emphasising again the importance of pre-specifying the range of thresholds of clinical relevance.
